# Supplementary material for: Novel Driver Strength Index highlights important cancer genes in TCGA PanCanAtlas patients
Source: PeerJ. 2022 Aug 11;10:e13860. doi: 10.7717/peerj.13860 (PMC9375969; doi:10.7717/peerj.13860)
Supplement: Supplemental Information 2 [file peerj-10-13860-s002.zip › COHORTS/genes plots/detailed/2021_8_16_14_9_distribution_events_detailed_females_10.pdf]

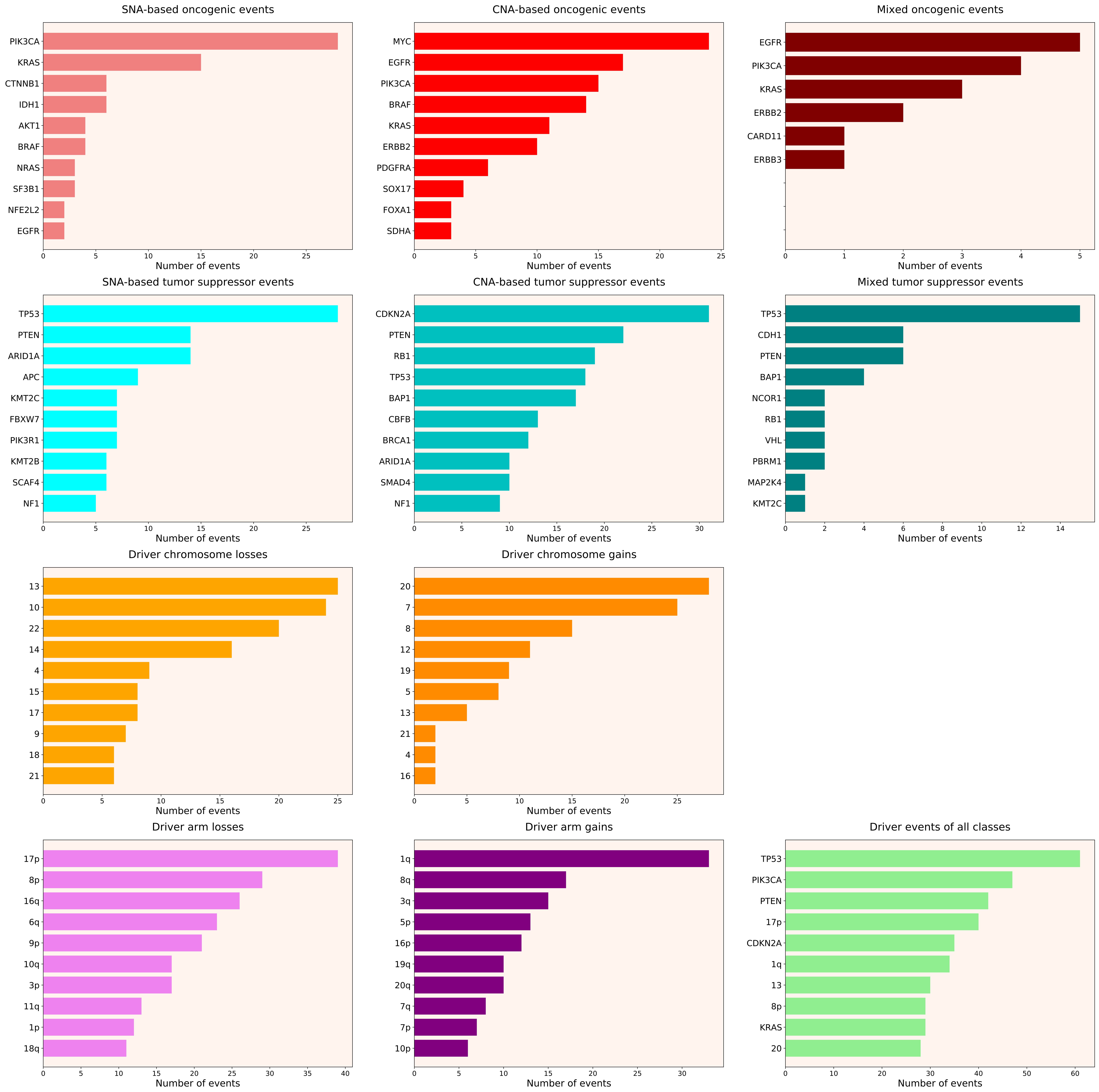

SNA-based oncogenic events
PIK3CA
KRAS
CTNNB1
IDH1
AKT1
BRAF
NRAS
SF3B1
NFE2L2
EGFR
Number of events
CNA-based oncogenic events
MYC
EGFR
PIK3CA
BRAF
KRAS
ERBB2
PDGFRA
SOX17
FOXA1
SDHA
Number of events
Mixed oncogenic events
EGFR
PIK3CA
KRAS
ERBB2
CARD11
ERBB3
Number of events
SNA-based tumor suppressor events
TP53
PTEN
ARID1A
APC
KMT2C
FBXW7
PIK3R1
KMT2B
SCAF4
NF1
Number of events
CNA-based tumor suppressor events
CDKN2A
PTEN
RB1
TP53
BAP1
CBFB
BRCA1
ARID1A
SMAD4
NF1
Number of events
Mixed tumor suppressor events
TP53
CDH1
PTEN
BAP1
NCOR1
RB1
VHL
PBRM1
MAP2K4
KMT2C
Number of events
Driver chromosome losses
13
10
22
14
4
15
17
9
18
21
Number of events
Driver chromosome gains
20
7
8
12
19
5
13
21
4
16
Number of events
Driver arm losses
17p
8p
16q
6q
9p
10q
3p
11q
1p
18q
Number of events
Driver arm gains
1q
8q
3q
5p
16p
19q
20q
7q
7p
10p
Number of events
Driver events of all classes
TP53
PIK3CA
PTEN
17p
CDKN2A
1q
13
8p
KRAS
20
Number of events
